# Native state structural and chemical characterisation of Pickering emulsions: A cryo‐electron microscopy study

**DOI:** 10.1111/jmi.13391

**Published:** 2025-01-31

**Authors:** Dario Luis Fernandez Ainaga, Teresa Roncal‐Herrero, Martha Ilett, Zabeada Aslam, Cheng Cheng, James P. Hitchcock, Olivier J. Cayre, Nicole Hondow

**Affiliations:** ^1^ School of Chemical and Process Engineering University of Leeds Leeds UK

**Keywords:** cryo‐electron tomography, cryogenic electron microscopy, cryo‐HAADF STEM, Pickering emulsions, plunge‐freezing, spectroscopy

## Abstract

Transmission electron microscopy can be used for the characterisation of a wide range of thin specimens, but soft matter and aqueous samples such as gels, nanoparticle dispersions, and emulsions will dry out and collapse under the microscope vacuum, therefore losing information on their native state and ultimately limiting the understanding of the sample.

This study examines commonly used techniques in transmission electron microscopy when applied to the characterisation of cryogenically frozen Pickering emulsion samples. Oil‐in‐water Pickering emulsions stabilised by 3 to 5 nm platinum nanoparticles were cryogenically frozen by plunge‐freezing into liquid ethane to retain the native structure of the system without inducing crystallisation of the droplet oil cores.

A comparison between the droplet morphology following different sample preparation methods has confirmed the effectiveness of using plunge‐freezing to prepare these samples. Scanning transmission electron microscopy imaging showed that dry droplets collapse under the microscope vacuum, changing their shape and size (average apparent diameter: ∼342 nm) compared to frozen samples (average diameter: ∼183 nm). Cryogenic electron tomography was used to collect additional information of the 3D shape and size of the emulsion droplets, and the position of the stabilising nanoparticles relative to the droplet surface. Cryogenic energy dispersive X‐ray and electron energy loss spectroscopy were used to successfully obtain elemental data and generate elemental maps to identify the stabilising nanoparticles and the oil phase. Elemental maps generated from spectral data were used in conjunction with electron tomography to obtain 3D information of the oil phase in the emulsion droplets.

Beam‐induced damage to the ice was the largest limiting factor to the sample characterisation, limiting the effective imaging resolution and signal‐to‐noise ratio, though careful consideration of the imaging parameters used allowed for the characterisation of the samples presented in this study. Ultimately this study shows that cryo‐methods are effective for the representative characterisation of Pickering emulsions.

## LAY DESCRIPTION

Among microscopy techniques, transmission electron microscopy boasts high‐resolution imaging for inorganic samples. However, in the case of soft matter where the natural state of the sample is hydrated, the vacuum in the microscope will affect the sample structure and therefore show an inaccurate representation of the sample. A commonly used solution to this issue in life sciences relies on freezing the sample before viewing in the microscope; this technique is however not fully explored when applied to inorganic materials. This study explores the application of commonly used techniques in transmission electron microscopy to cryogenically frozen samples (specifically, Pickering emulsions) to understand the advantages and limitations of this sample preparation method. A variety of imaging and elemental analysis techniques were successfully applied to obtain 2D and 3D information on the structure and elemental composition of the sample and were discussed in terms of their suitability for cryogenically frozen samples.

## 1 INTRODUCTION

Transmission electron microscopy (TEM) is routinely used for the characterisation of nanomaterial‐based systems due to the high magnification and resolution imaging, alongside associated spectroscopy techniques that are possible. Routine imaging and analysis, however, does limit specimens by both size and stability under the vacuum of the electron microscope.

This creates challenges when imaging soft matter and aqueous samples such as nanoparticle dispersions and emulsions as imaging at high resolution using TEM can lead to the loss of sample structure information, such as particle agglomeration for example, due to drying artefacts.^1^, ^2^ Consequently, representative characterisation of the sample in its native state (i.e. its original aqueous state, prior to any sample preparation needed before imaging) is not possible.^3^ Alternative methods for sample preparation such as staining the sample with a metallic component or stabilising with a polymer have been previously used.^4^, ^5^ However, while these techniques can provide some useful information, neither method is successful in fully preserving the original sample morphology.

Cryogenic (cryo)‐fixation techniques (e.g. plunge‐freezing or high‐pressure freezing followed by cryo‐ultramicrotomy) provide a route for the TEM imaging of aqueous samples while maintaining their native morphology and local concentration. These techniques were developed by Dubochet et al. in the 1980s focusing on the use of cryo‐EM to study viruses. Alongside, extensive studies were carried out by Talmon et al. looking at cryo‐EM of soft condensed matter, lipids and surfactants.^6^, ^7^, ^8^, ^9^, ^10^ Since then, cryo‐EM has been routinely used within the life sciences and there have been significant advancements made in the technologies and methodologies used.^11^, ^12^ While these methods have been used and developed for many years within the life sciences they are now becoming successfully applied to the preparation of nonbiological samples like particle dispersions (e.g. nanoparticles within a commercial sunscreen), nanotubes and emulsions among others.^13^, ^14^, ^15^, ^16^, ^17^ Cryo preparation is ideal for such systems due to the vitrification of liquid phases that provides the optimum conditions for observation of hydrated species in their native state since the first‐order exothermic phase transition between the liquid and the solids does not occur.^18^ Even after successful sample vitrification, it is important to be aware of the homogeneity across the grid and ensuring the ice is appropriately thin to allow electron transmission.

A specific type of emulsion are Pickering emulsions, which are systems composed of one liquid in the form of droplets dispersed within another immiscible liquid and stabilised by solid particles. Unlike conventional emulsions stabilised by surfactants, Pickering emulsions use solid particles adsorbed at the liquid‐liquid interface that stabilise the system through a combination of steric effects and capillary forces.^19^, ^20^, ^21^, ^22^ The stability of these emulsions is related to the detachment energy of the stabilising particles, since particles must desorb from the interface to facilitate coalescence of the droplets into a fully separated system. Understanding and controlling the distribution of the solid particles between the bulk and the interface is essential for the manufacture of Pickering emulsions, which can be used in a wide range of application areas including lubrication, food engineering, and drug delivery.^20^, ^23^, ^24^, ^25^

Pickering emulsions are traditionally characterised in the native state using dynamic light scattering (DLS) and light microscopy to obtain information of the size, shape, and agglomeration state of the emulsion droplets and stabilising particles.^26^, ^27^, ^28^, ^29^, ^30^ However, the characterisation of smaller components in these systems is limited by the resolution of these techniques; emulsions stabilised with smaller nanoparticles (<100 nm) and small emulsion droplets (emulsions with droplet size <1 µm) require higher resolution when sample imaging is required. AFM and electron microscopy have been used to characterise dry samples in cases when only information of the stabilising particles was needed.^22^, ^31^, ^32^, ^33^ Sample imaging in the native state, that is, when the droplet‐based system is dispersed in liquid, can be accomplished through extensive sample preparation. This includes fixation, freeze‐fracture and plunge‐freezing.^27^, ^34^, ^35^, ^36^, ^37^ These techniques can be useful when attempting to characterise the distribution and position of the particles within the system and any possible aggregation as these factors will affect the stability of the system. Silva et al. (2020) used light microscopy, AFM, and cryo‐TEM to characterise Pickering emulsions in their native state, with cryo‐TEM specifically being used to analyse the dispersion of stabilising cellulose nanofibres.^27^ A range of cryo‐EM imaging techniques are often used in conjunction with light microscopy or other techniques for the characterisation of Pickering emulsions, such as freeze fracture used by Chen et al. (2011) and cryo‐scanning electron microscopy used by Xiao et al. (2016).^26^, ^38^

Among these techniques, cryo‐TEM allows for not only direct imaging of the Pickering emulsion samples in their native state, but also allows for a wider range of characterisation techniques to be used to complement the imaging, including electron energy loss spectroscopy (EELS) and energy dispersive X‐ray spectroscopy (EDX), which provide additional elemental information for the characterisation of stabilising particles and droplet phase.^39^, ^40^, ^41^

3D characterisation techniques provide important information on the size, shape, and agglomeration of emulsion droplets that would otherwise be unattainable with conventional imaging techniques. To achieve this, tomography, that is, collection of a tilt series followed by 3D reconstruction, can be used together with different microscopy techniques including electron microscopy.^42^, ^43^

This study examines a range of transmission electron microscopy approaches for the characterisation of platinum nanoparticle stabilised oil‐in‐water Pickering emulsion samples. The main aim of this study is to explore the use of cryo‐(S)TEM, ‐tomography, and ‐spectroscopy for the representative characterisation of Pickering emulsions in their native state, in particular to obtain specific information of the droplet interface in the nanoscale. Bulk characterisation of the emulsion samples was first performed using DLS to measure the emulsion droplet size in the native state. Samples were then imaged in TEM both in their dry state and when cryogenically frozen using conventional TEM and high angle annular dark field scanning transmission electron microscopy (HAADF STEM) to analyse the changes in droplet shape and size resulting from the sample preparation methods. Electron tomography was used to obtain further information on the shape and distribution of the emulsion droplets in 3D space. Furthermore, EELS and EDX were used to provide spectral information related to the dispersed oil phase and stabilising particles.

## 2 MATERIALS AND METHODS

### 2.1 Pickering emulsion

Two similar Pickering emulsions were examined: a sunflower oil‐in‐water emulsion stabilised by 3 to 5 nm diameter platinum nanoparticles (Pt‐NPs); and a hexadecane‐in‐water emulsion stabilised by the same 3 to 5 nm Pt‐NPs. The oils have broadly similar properties as far as their behaviour in these emulsions are concerned. The sunflower oil emulsion was prepared by first hand‐shaking a 20 mL sample tube containing 2 mL of oil and 8 mL of the pre‐prepared Pt‐NPs aqueous suspension. The emulsion was then immediately prepared using a sonic dismembrator ultrasonic processor (Fisher Scientific) for 1 min (40% amplitude) in a large water bath (at room temperature) to avoid dramatic increase of the sample temperature. The hexadecane emulsion was prepared by hand‐shaking a 10 mL sample tube containing 20 µL of hexadecane oil and 4.98 mL of Pt‐NPs in suspension. This resulted in an emulsion with 0.04 vol% oil concentration. The same Pt‐NPs were used in both samples and were synthesised by the reduction of platinum salt in the presence of a reducing agent at room temperature as detailed elsewhere.^25^

### 2.2 TEM sample preparation

For conventional, dry TEM observations, 3.5 µL of a freshly inverted Pickering emulsion sample was deposited onto a plasma cleaned lacey carbon film supported on 400 mesh copper grids (EM Resolutions) or onto a SiNx grid (EM Resolutions). For cryo‐TEM observations lacey carbon films supported on 200 mesh copper grids were plasma cleaned for 20 s and 3.5 µL of the freshly inverted Pickering emulsion sample was deposited using an FEI Vitrobot© mark IV plunge freezer. The sample was blotted for 4 s at blot force 6 and then plunged into liquid ethane. The sample was transferred into a Gatan 914 cryo‐holder for TEM and the temperature maintained below –170°C to prevent devitrification.

### 2.3 Cryo‐TEM

A FEI Titan Themis^3^ TEM operated at 300 kV with a monochromator, multiple STEM detectors, Gatan One‐View CCD, Super‐X 4‐detector silicon drift EDX system and a Gatan GIF Quantum 965 EELS was used. The monochromator allowed continuous beam current control in STEM when not excited and the X‐FEG source provided an energy spread of ∼ 1.1 eV (FWHM of zero loss peak) in this condition. STEM was run with a 1.4 Å probe diameter of 10 mrad convergence semi angle. HAADF images were collected over the scattering semi‐angle range of 35–150 mrad. STEM‐EEL spectra were collected with a 20.2 mrad collection semi‐angle and were analysed using the Gatan DigitalMicrograph software and Hyperspy.^44^ Probe currents were varied using the monochromator and ranged from 5 to 100 pA depending on the imaging and mapping mode. EDX spectra were processed in VELOX.

### 2.4 Cryo‐STEM tomography

A tilt series of a region of interest was taken by collecting images using the FEI STEM Tomography software in cryo‐HAADF STEM mode with a probe current of 5 to 10 pA. A frame was collected every 3° from α = –60° to α = +60° with a pixel resolution of 1.8 nm/pixel. The tilt series was reconstructed using simultaneous iterative reconstruction technique (SIRT) after being processed and aligned in the Inspect3D software. Renders of the reconstructed tomogram were generated after adding surfaces to represent the oil droplets and Pt‐NPs using the Imaris software (version 9.5.0). Surfaces for the droplets were drawn manually using the position of the stabilising NPs as a reference outline and are therefore only an approximation of the droplet. The Pt‐NPs were instead represented using the automatic spot creation function in Imaris, then manually adjusted using the original cryo‐HAADF STEM images as reference.

### 2.5 Image analysis

The shape of the emulsion droplets was measured using ImageJ in a population of 500 emulsion droplets.^45^ Using the STEM images, the shape of the droplets was underlined and an ellipsoid was adjusted to the shape. In this way, the diameter of the emulsion has been chosen to be the major axis of the ellipsoid. The circularity is defined by Equation (1).

(1) $$\mathrm{Circ}=4\pi \;\frac{\mathrm{Area}}{\mathrm{Perimete}r^{2}}.$$

The circularity ranges from 0 (infinitely elongated polygon) to 1 (perfect circle). Density of the Pt nanoparticles at the emulsion droplet was calculated by counting the number of Pt nanoparticles present in the central area of the spherical projection in an area equivalent to 1/3 of the diameter. Although a change in focus can be observed due to the curvature of the droplet, the particles could still be counted reliably.

## 3 RESULTS AND DISCUSSION

### 3.1 Bulk analysis and initial electron microscopy

Optical light microscopy is one of the simplest methods to visualise emulsions and was used in our previous work to confirm the droplet size and shape of the Pt‐NPs‐stabilised Pickering emulsions studied here.^18^ However, the observations are limited due to the possible spatial resolution restricting the ability to image the smaller droplets (and the stabilising NPs cannot be resolved).^25^ These measurements may also find difficulties due to destabilisation of the emulsion resulting in coalescence and Ostwald ripening or Brownian motion movement on the cover slips when these phenomena are not accounted for.^46^, ^47^ For the samples presented here, the smallest droplets were too small to allow for representative characterisation using a light microscope and as would be expected, this method does not provide information on the Pt particles or their distribution on the Pickering emulsion droplets. In addition to light microscopy, DLS was utilised to measure the size distribution of the emulsion droplets, with the mean diameter observed to be ∼100 nm for both sunflower oil and hexadecane emulsions as seen in the number distribution DLS plot (Figures S1 and S2). However, DLS itself is limited as it measures only the hydrodynamic diameter and therefore the technique cannot distinguish between the emulsion droplets and stabilising particles.

**FIGURE 1:**
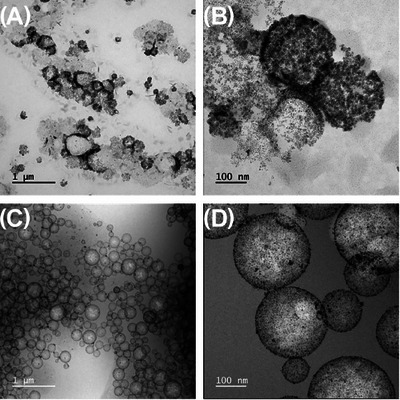
Comparison of Pt‐NP‐stabilised oil‐in‐water Pickering emulsion characterisation using TEM and cryo‐TEM showing a general view of two preparation methods for the Pickering emulsion sample. In (A) and (B), the sample was drop‐cast and air‐dried, showing emulsion droplets that burst under the electron microscope vacuum. In (C) and (D), the sample was plunge‐frozen and imaged in cryo‐TEM with a total electron fluence of (C) 41 e^−^/Å^2^ and (D) 781 e^−^/Å^2^ and shows that cryogenic preparation was successful in preserving the droplets. Although the sample used is the same across all four images, the preparation method for EM characterisation greatly influences the preservation of the sample morphology.

Electron microscopy has been identified as an important technique to break through this resolution limit in cases where the stabilising nanoparticles need to be imaged.^31^ However, conventional electron microscopy is prone to artefacts as seen in surfactant solutions, hydrated colloidal dispersions and biological samples.^48^ Drop‐casting, where a drop of the sample is placed onto a TEM grid and left to air dry, can cause drying artefacts, modifying the morphology and structure of the emulsion due to evaporation of the liquid phase. Moreover, if some of the droplets were kept intact, the vacuum in the microscope would cause shrinkage, collapse and dehydration of the sample.^1^, ^49^ These drying artefacts are seen in the drop‐cast TEM images in Figure 1A and B. Under the vacuum of the TEM, the emulsion droplets have burst, with a contour marked by the Pt‐NPs and with a variable morphology and size that is not in agreement with the DLS data (Figure S1), which is expected from a dry sample. EDX of the dry sample shows the presence of the platinum signal from the collapsed emulsion droplets, but the lack of a distinct carbon peak overlapping with the Pt signal suggests, as expected, that the oil phase is not contained within a spherical droplet, due to collapse, and subsequent evaporation, in the vacuum of the electron microscope.^50^

### 3.2 Cryogenic transmission electron microscopy

Having shown a loss of structural integrity of the emulsion droplets when prepared via drop‐casting for TEM analysis, cryo‐TEM was investigated as an alternative technique that allows the observation of samples in their hydrated state. Electron fluence (the total number of electrons incident on the sample per unit area) and dose (the rate of electrons per unit area) are critical parameters for cryo‐EM observation. A minimum amount of electrons is required to generate enough contrast, while above a critical electron fluence, significant sample damage will occur. Dose is likewise important to effectively manage the electron fluence before and during imaging; critically, this has impact on steps such as sample navigation and focusing.

**FIGURE 2:**
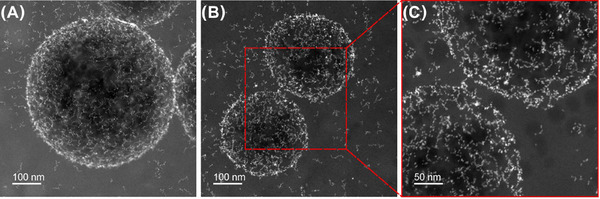
(A) Size distribution of 500 Pickering emulsion droplets measured using TEM images at various magnifications and both after drying and of cryo‐preserved samples and (B) circularity and diameter of the 500 emulsion droplets measured. As it can be seen in Figure 1, the droplet morphology tends to change as the droplets dry and collapse. Droplets analysed by the drop‐cast method range from 98 to 1089 nm, with an average size of ∼342 nm. Cryogenically frozen droplets range from 45 to 514 nm, with an average size of ∼183 nm.

Cryogenic preparation of the Pickering emulsions followed by imaging using appropriate electron fluence and doses showed significant differences compared to the drop‐cast samples where the oil droplets maintained their original spherical morphology following vitrification (Figures 1 and 2). In addition cryo‐analytical STEM was used alongside conventional TEM as previous work by Ilett et al. (2019) and Egerton (2019) has demonstrated that the damage due to the electron beam in cryo‐analytical STEM occurs at higher electron fluences (<2000 e^−^/Å^2^) as compared to conventional cryo‐TEM (<100 e^−^/Å^2^).^39^, ^51^ This is due to diffusion limited damage by the radiolysis products generated in vitreous ice.^39^ Furthermore, cryo‐HADDF STEM is useful when imaging this Pickering emulsion system due to atomic number contrast which is highlighted in these samples with high atomic number Pt‐NPs in low atomic number oil and water (Figure 2).

**FIGURE 3:**
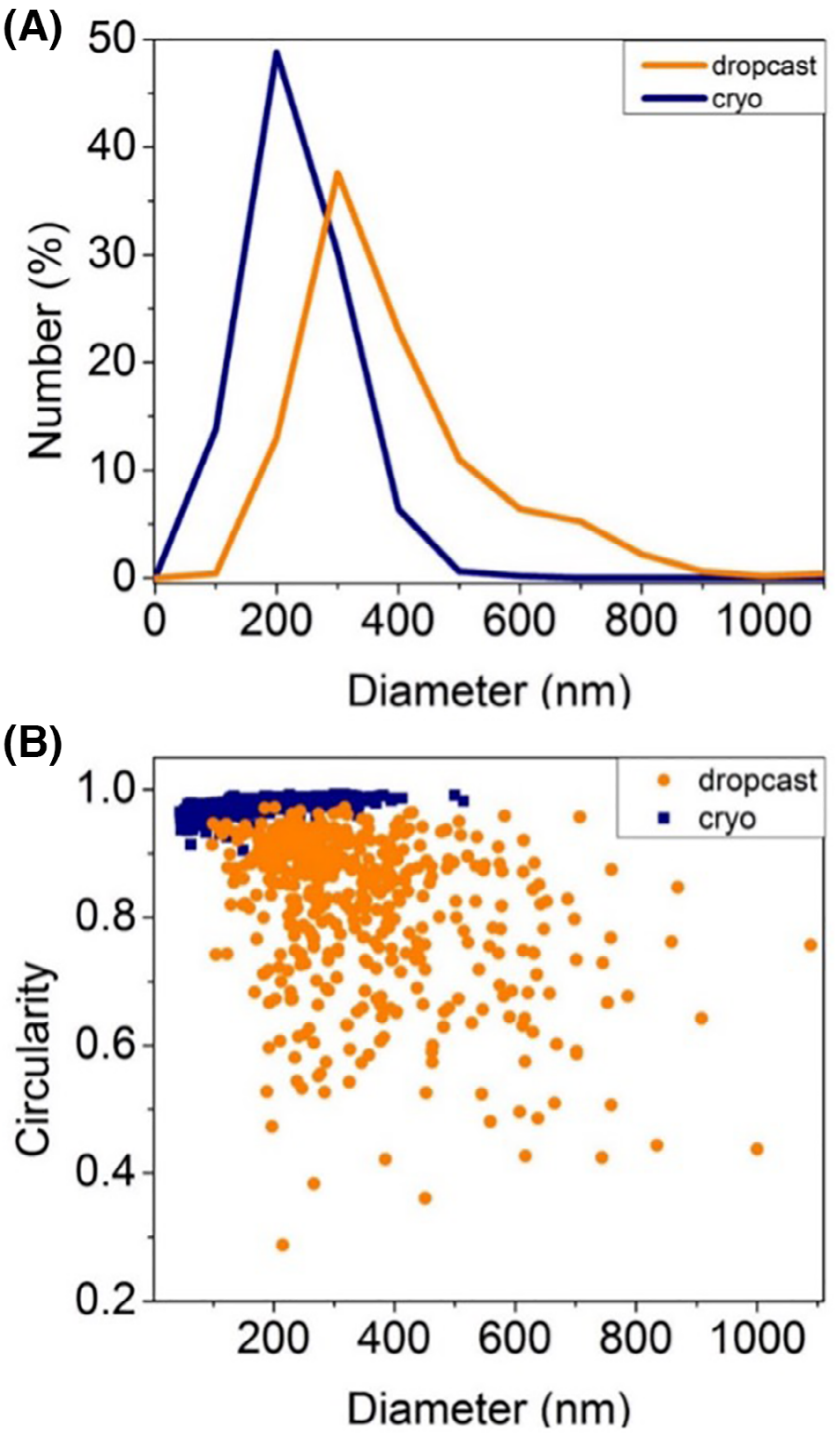
Cryo‐HAADF STEM images of the hexadecane Pt‐Pickering emulsion sample with total electron fluences of (A) 63 e^−^/Å^2^, (B) 66 e^−^/Å^2^ and (C) 267 e^−^/Å^2^. It was possible to obtain higher magnification images where the 3 to 5 nm Pt‐NPs could be resolved as seen in (C) due to a higher total electron fluence being required to cause beam damage when using STEM.

The emulsion droplets imaged are circular, implying the spherical shape of the droplets is maintained within the vitreous ice. The average diameter of 500 droplets measured from the drop‐cast samples was 340 nm (ranging from 98 to 1089 nm) and for the cryo‐prepared sample was 183 nm (ranging from 45 to 514 nm) (Figure 3). The variability on the diameter measurements is extreme, with a measured mean diameter from drop‐cast STEM measurements much greater than the bulk DLS measurement (Figure S1), and a slight increase in the mean diameter measured from the cryo‐prepared sample compared to DLS. The information on emulsion size and distribution is a critical factor for the design of materials and its application and therefore bulk measurement should be interpreted carefully. The collapse of the oil droplets in the drop‐cast sample is evident through the decrease in circularity as compared to the cryo‐preparation (Figure 3B). This is consistent with Figure 1A and B where the emulsion droplets burst and collapsed during drop‐cast and air‐drying on a TEM grid. As such, for samples prepared via drop‐casting the circularity ranges from 2.82 to 1.01 and for the cryo‐prepared sample between 1.22 and 1.01 (Figure 3B).

These results suggest that the cryo‐prepared sample retains both the droplet size and shape that would be expected in its aqueous state. The larger range in the droplet circularity in the drop‐cast sample is reflected in the (S)TEM images of collapsed droplets shown in Figure 1A and B. However, this analysis is limited to two dimensions and cannot detect deformation in the direction normal to the viewing plane as a result from blotting the sample during plunge‐freezing. The flattening of large samples was also observed by Wills et al. (2017) in nanoparticle dispersions cryogenically frozen by plunge‐freezing and subsequently dried under a high vacuum, where agglomerates were shown to be flattened.^52^ This issue was tackled by acquiring images at multiple tilt angles and creating a tomogram of the interested area to observe it in 3D and therefore confirm that the droplet shape is maintained through the sample preparation steps. The use of tomography here comes from applying developments already present and used in the life sciences where cryo‐EM tomography is a common technique used in studying proteins and cells.^53^, ^54^, ^55^ Drawing from these advances it is now becoming a valuable technique within materials research.

Plunge‐freezing and cryo‐(S)TEM imaging were successfully applied for the analysis of this Pickering emulsions sample, being able to image individual droplets, small droplet clusters, and Pt‐NPs. Large droplet aggregates were also found near the grid bars but were often not suitable to imaging due to the excessive ice thickness. The presence of larger droplets and droplet aggregates (∼600 nm) was also confirmed from Figure S2, where the DLS size distribution by volume shows a peak at ∼600 nm diameter. However, cryo‐(S)TEM and plunge‐freezing are not suitable for the analysis of large droplets (>600 nm diameter) or droplet aggregates because these may be affected by the sample preparation and are largely inaccessible for cryo‐(S)TEM imaging as they are found preferentially in area with thick, crystalline ice. For Pickering emulsion samples with large droplet sizes, or when the analysis of droplet aggregates is important, other techniques should be used (e.g. DLS, optical microscopy, cryo‐scanning electron microscopy).

### 3.3 3D visualisation using cryogenic electron tomography

To verify the hypothesis that the emulsion droplets maintain their original 3D shape through the blotting stage, a cryo‐HAADF STEM tomography tilt series was collected from –60° to +60° taking a frame every 3°. The tilt series was collected in cryo‐HAADF STEM mode to mitigate the beam‐induced damage that accumulates as images are taken.^56^ Nonetheless, the overall quality of the tilt series is greatly limited by cumulative damage suffered by the sample. This damage can be controlled by changing the beam current, dwell time, pixel size, and number of tilt steps: lowering the beam current and dwell time will decrease the electron fluence but will also lower the signal. Increasing the pixel size will likewise decrease the electron fluence but decrease the image resolution. Lastly, the number of tilt steps can be decreased, thus decreasing the number of images that need to be taken per tilt series, but this also affects the quality of the 3D reconstruction.^57^, ^58^, ^59^ In addition to mitigating beam‐induced damage, using HAADF STEM for tomography is advantageous as it will limit any diffraction contrast that would otherwise be evident in cryo‐TEM imaging and therefore limit any 3D reconstruction.

**FIGURE 4:**
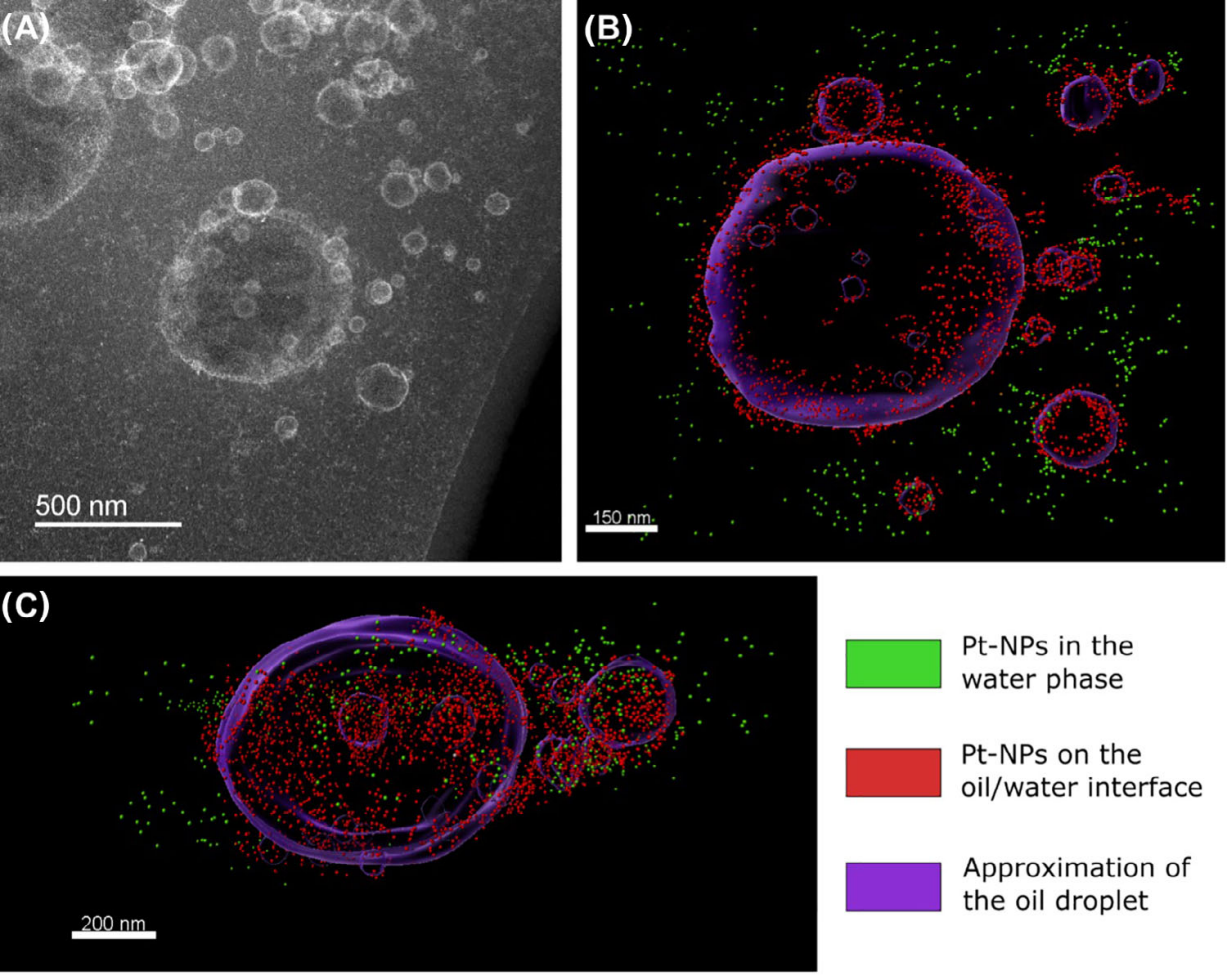
Cryo electron tomogram and corresponding cryo‐HAADF STEM image of droplets. (A) Cryo‐HAADF STEM image; (B, C) top‐view and side‐view of the of an electron tomogram taken from the area shown in (A). Although it was not possible to use a magnification high enough to consistently resolve the individual Pt‐NPs without completely damaging the sample, the position of NP chains in 3D space is still visible in the tomogram. Total electron fluence: (A) 67 e^−^/Å^2^, electron tomogram 1688 e^−^/Å^2^.

A tilt series comprised of a total of 41 images (an example of which is shown in Figure 4, full tilt series in Video S1) was collected using a pixel size of 1.8 nm and a probe current of 10 pA. A tomogram was reconstructed from the tilt series using SIRT after being aligned in the Inspect3D software. Due to the low contrast for the oil phase, the 3D surfaces for the droplets were created manually and are therefore only an approximation (Figure S3). The completed 3D reconstruction in Figure 4 shows the position of the emulsion droplets and Pt‐NPs. Figure 4C shows the three‐dimensional nature of the droplets is retained after cryogenic preparation, as the droplets have not collapsed. The Pt‐NPs are distributed mainly along the oil–water interface, but also present in the water phase on the surface of the ice. The presence of Pt‐NPs on the surface of the vitreous ice might be an artefact of the sample preparation arising from the movement of the nanoparticles before the sample is plunged into the liquid ethane but it is also possible that these nanoparticles are not adsorbed to the oil–water interface and remain in the aqueous continuous phase in the emulsion sample. The former effect has been observed in inorganic samples with nanoparticle markers being found at the surface of the cryogenically‐frozen sample, and in biological samples, where the effect of the interface can induce a bias on the direction of cell, viruses, and particles, and therefore affect their characterisation.^60^, ^61^, ^62^

The size of the Pt‐NPs was the main limiting factor for controlling the amount of beam damage, as the need to resolve the 3 to 5 nm nanoparticles affected the minimum pixel size and magnification needed to image both the nanoparticles and oil droplets.^63^ The effects of this issue can be seen in Figure 4A and B: in Figure 4B only clusters of Pt‐NPs can be seen as the image resolution for the tilt series and resulting 3D reconstruction had to be decreased to limit beam damage. STEM pixel size and dwell time are not the only parameters to be considered when collecting a tilt series in cryo‐(S)TEM: not only is the number of tilt steps in a series a major contributor to the total electron fluence, but so are the focusing and tracking steps (Figure S4). Tilt tracking can be accomplished with images with low pixel resolution, therefore accounting for only a small portion of the total electron fluence. Sample focusing can be approached by either directly focusing on the area of interest or by moving the beam to focus on a nearby feature. Neither strategy can be thought as a catch‐all solution for this system, as the former allows for accurate focusing but is more energy intensive, while the latter fully prevents damage to the area of interest but requires the presence of nearby features along the tilt axis. This requirement is often not met, as in this system droplets found in clusters tend to be near thicker ice, and therefore less suitable for collecting a tilt series.

The 3D reconstruction results obtained show that HAADF STEM imaging with atomic number contrast allows for straightforward analysis of the Pt‐NPs in 3D space. However, in this imaging mode the oil phase does not generate enough contrast to be successfully imaged and it is therefore not possible to locate the position of the oil–water interface.

### 3.4 Cryo‐spectroscopy EELS and EDX analysis

**FIGURE 5:**
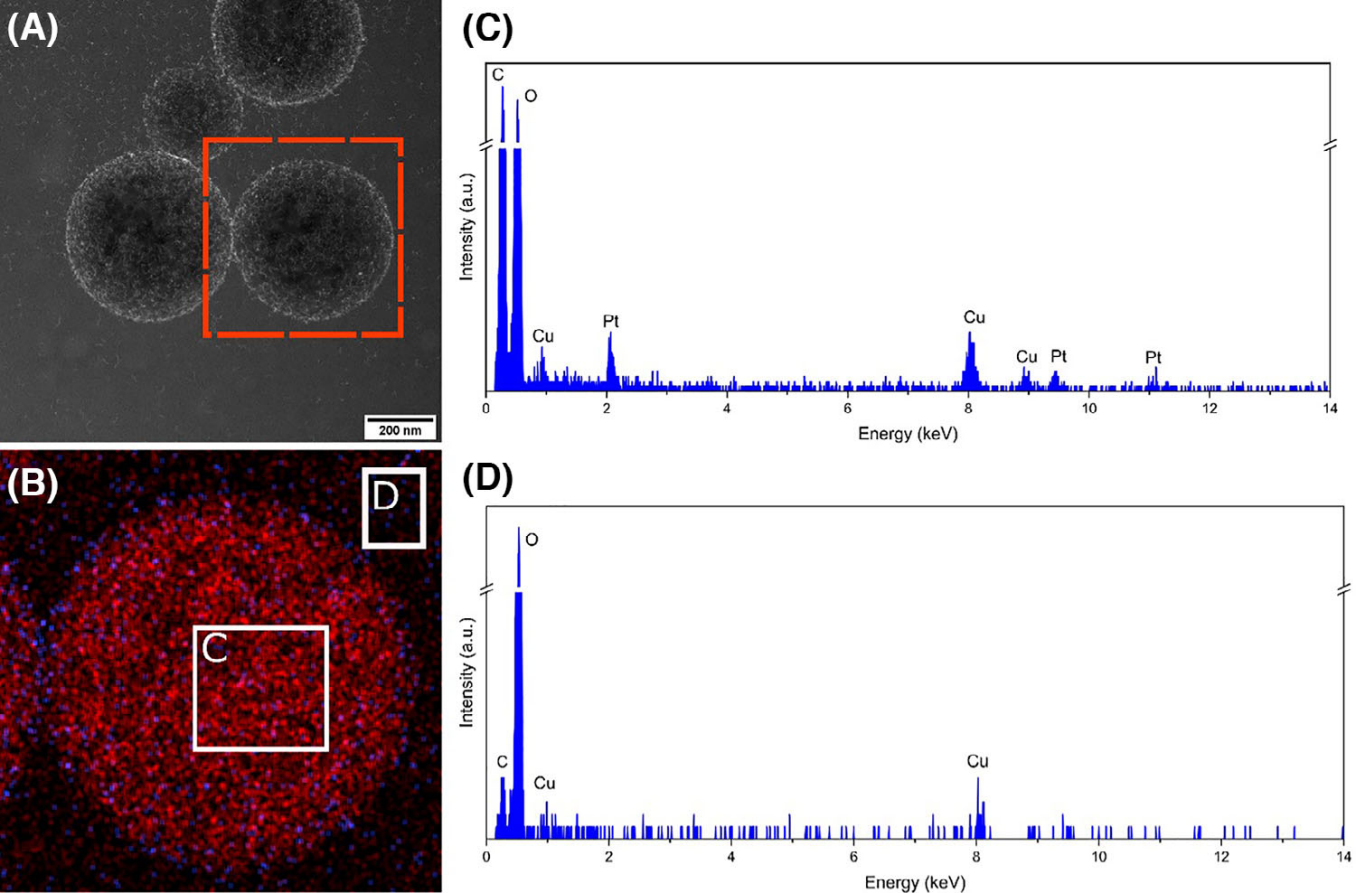
(A) HAADF‐STEM images of a Pt‐NPs‐stabilised Pickering emulsion plunge‐frozen onto lacey carbon grid and (B) EDX elemental map showing the carbon (red) and platinum (blue) signals with boxes showing EDX spectrum locations. (C, D) Corresponding EDX spectra (total electron fluence: 732 e^−^/Å).

Elemental analysis of the frozen sample was performed using EELS and EDX mapping while in HAADF‐STEM mode to provide information about both the presence of the oil phase within the emulsion droplets and the location of the stabilising Pt‐NPs. The EDX elemental map of the cryo‐prepared sample in Figure 5B shows a carbon signal within the droplet corresponding to the oil phase and a platinum signal localised predominantly on the droplet interface. A challenge for the elemental analysis of this oil phase lies in distinguishing the carbon signal corresponding to the oil from the surroundings (e.g. the carbon support film). To resolve this issue, elemental analysis of the frozen sample was only performed on droplets found over holes in the carbon film; the drop‐cast sample was instead imaged on a SiNx grid to prevent the carbon signal from conventional carbon TEM grids which would otherwise overlap with the carbon signal from the oil phase.

**FIGURE 6:**
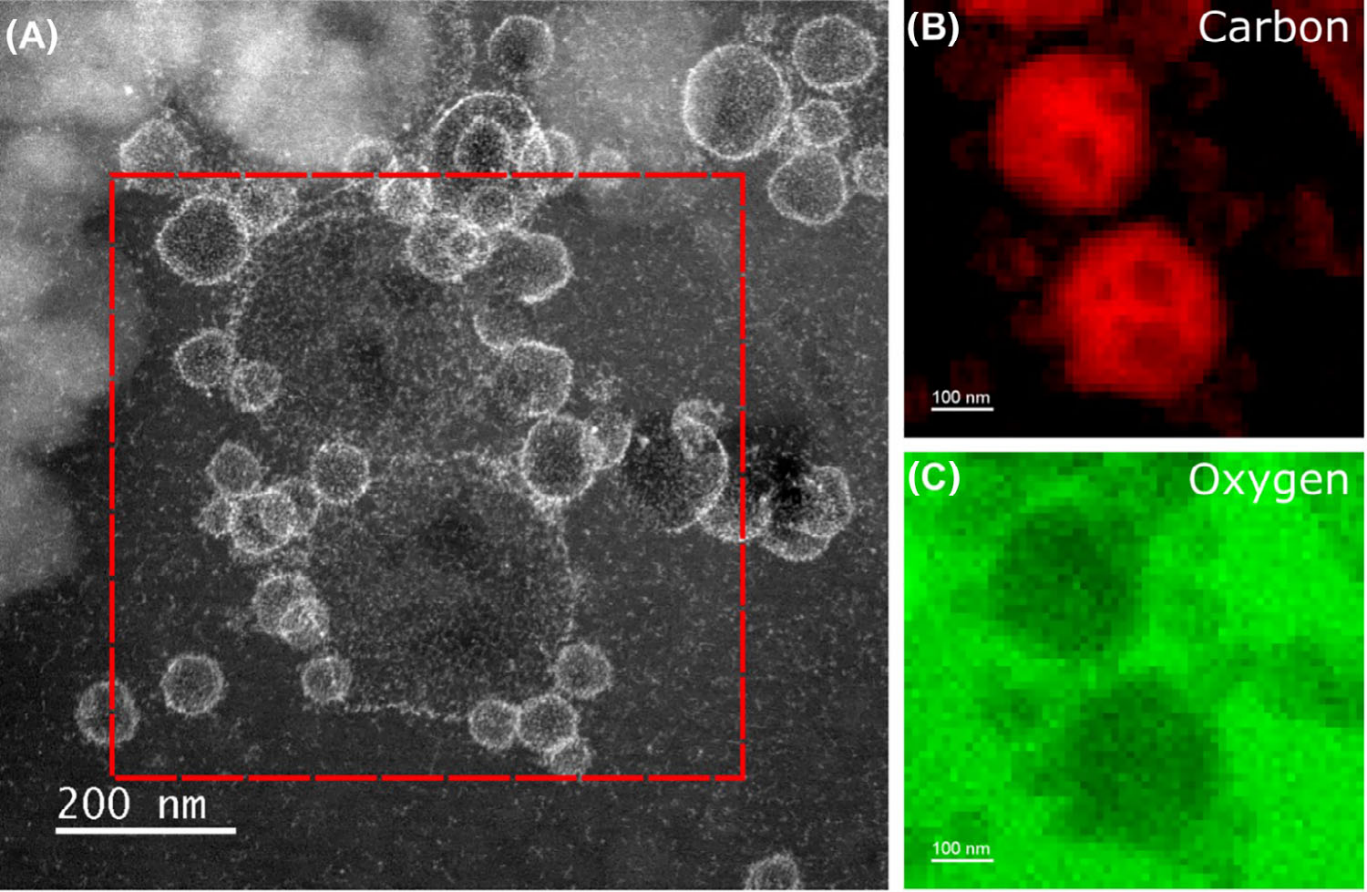
EELS elemental maps of emulsion droplets in the Pickering emulsion sample. (A) Cryo‐HAADF STEM of emulsion droplets (total electron fluence of 16 e^−^/Å^2^). (B, C) Maps of the signal intensity for the C K and O K edges respectively (16 e^−^/Å^2^).

EELS elemental maps (Figure 6) were collected to support the results from EDX analysis. In the EEL spectrum imaging, detection of the carbon and oxygen edges were focused on in order to detect the carbon oil phase and the oxygen in the vitreous ice, therefore permitting understanding of both the presence of the oil in the droplets and also what proportion of the ice thickness they occupy. The oxygen and carbon K edges are both found at a relatively low energy loss and are therefore possible to collect using a low total electron fluence.

The EELS maps were taken with a 0.09 s exposure time and 12 nm pixel size (resulting in a total electron fluence of 16 e^−^/ Å^2^), as an increase in exposure time or decrease in pixel size would result in an increase in electron fluence which would then cause damage to the sample. EELS exposure time and pixel size will need to be adjusted on a sample‐to‐sample basis depending on sample size, elements present in the sample, and damage threshold for the specific sample.

The lower electron fluence needed to obtain the elemental map when compared to EDX spectroscopy is especially attractive for cryo‐STEM imaging, as it can not only allow for the use of multiple techniques to characterise areas of interest but could also be used in conjunction with cryo‐Electron Tomography (cryo‐ET) to generate 3D objects based on the elemental maps without damaging the sample. In cryo‐STEM the elemental analysis provided by EELS is limited by the presence of important major edges at high energy losses, as is the case with the Pt major edges which cannot be accessed without sample damage. However, Pickering emulsion may be stabilised by a range of different particles and depending on the elemental composition of these particles, it may be possible to analyse them using EELS. For example, silica NPs may be more readily analysed using EELS due to silicon having more accessible L‐edges near 100 eV.

So far, neither EDX nor EELS can be thought of as a catch‐all for elemental analysis of these Pickering emulsion samples. EELS can provide fast, single‐pass, and low‐dose elemental maps that can be used to quickly analyse elements within a limited electron energy loss range. For the Pickering emulsion sample used here, the carbon and oxygen K‐edges can be easily collected, but the major platinum edges above 2000 eV are outside of the safe detection range to prevent damage to the sample. A longer exposure time would be needed to detect the platinum signal in this system, possibly in the order of 1–10 s, therefore increasing the overall fluence by a factor of 100 before taking into consideration any increased damage from decreasing the pixel size to resolve individual nanoparticles. On the other hand, EDX can be used to detect most elements despite the lower energy resolution. The 3 to 5 nm Pt‐NPs used in these samples are challenging to analyse, with EDX being unable to distinguish between individual NPs, and EELS being unable to reach a pixel size below 12 nm in the microscope setup used without significant damage that irreversibly changes the sample.

The absolute thickness of the sample was estimated from the collected EEL low‐loss spectra using Hyperspy as shown in Figure S5.^44^ Areas with no sample present (Figure S5B) and areas with emulsion droplets (Figure S5C) were both analysed to ensure that the ice thickness is sufficient to contain the emulsion droplets without deformation. The amorphous ice within holes in the carbon film was estimated to be approximately ∼250 nm thick near the centre of the grid squares, with thickness variation present near the carbon film and approaching the copper grid. This absolute thickness calculation was done using a mean inelastic free path for vitreous ice of ∼350 nm.^60^ This result can be contrasted with Figure S5C, where the ice thickness was estimated to reach up to ∼500 nm near clusters of emulsion droplets. Plunge‐freezing cryo‐TEM might therefore be suitable for the imaging of droplets and clusters up to a few hundreds of nanometres (<600 nm) in diameter without deformation or damage to the droplets. However, cryo‐(S)TEM imaging can still allow for the imaging of larger droplet aggregates trapped within the ice with the only imaging limitation being given by the presence of thicker ice near the grid bars which decreases the signal‐to‐noise ratio when imaging and increases the likelihood of crystalline ice forming during the sample preparation. For EELS analysis the droplet size limitation is more stringent: a minimum droplet size of ∼50 nm could be analysed with the methods presented in this study, limited by the smallest pixel size available before beam‐induced damage begins accumulating. Identifying an upper limit for EELS can be more challenging, as it is dependent on the information that needs to be extracted. For low‐loss EELS analysis and thickness estimation, the same limits as imaging apply due to the ice being generally thinner than 2–3 times than its mean inelastic free path. However, techniques like EELS mapping and elemental quantification cannot be applied to areas where the sample thickness is much thicker than the mean inelastic free path, therefore only allowing for the analysis of single droplets or small aggregates up to 400–500 nm in diameter. Electron tomography can be applied for the imaging of large aggregates (similarly to regular cryo‐(S)TEM imaging) but can only be used for samples near the centre of the grid square, as the grid bars can obstruct the sample at high tilt angles. Moreover, care needs to be taken when imaging the sample at high tilt angles as the effective sample thickness increases together with the tilt angle.

### 3.5 Electron tomography with EELS

**FIGURE 7:**
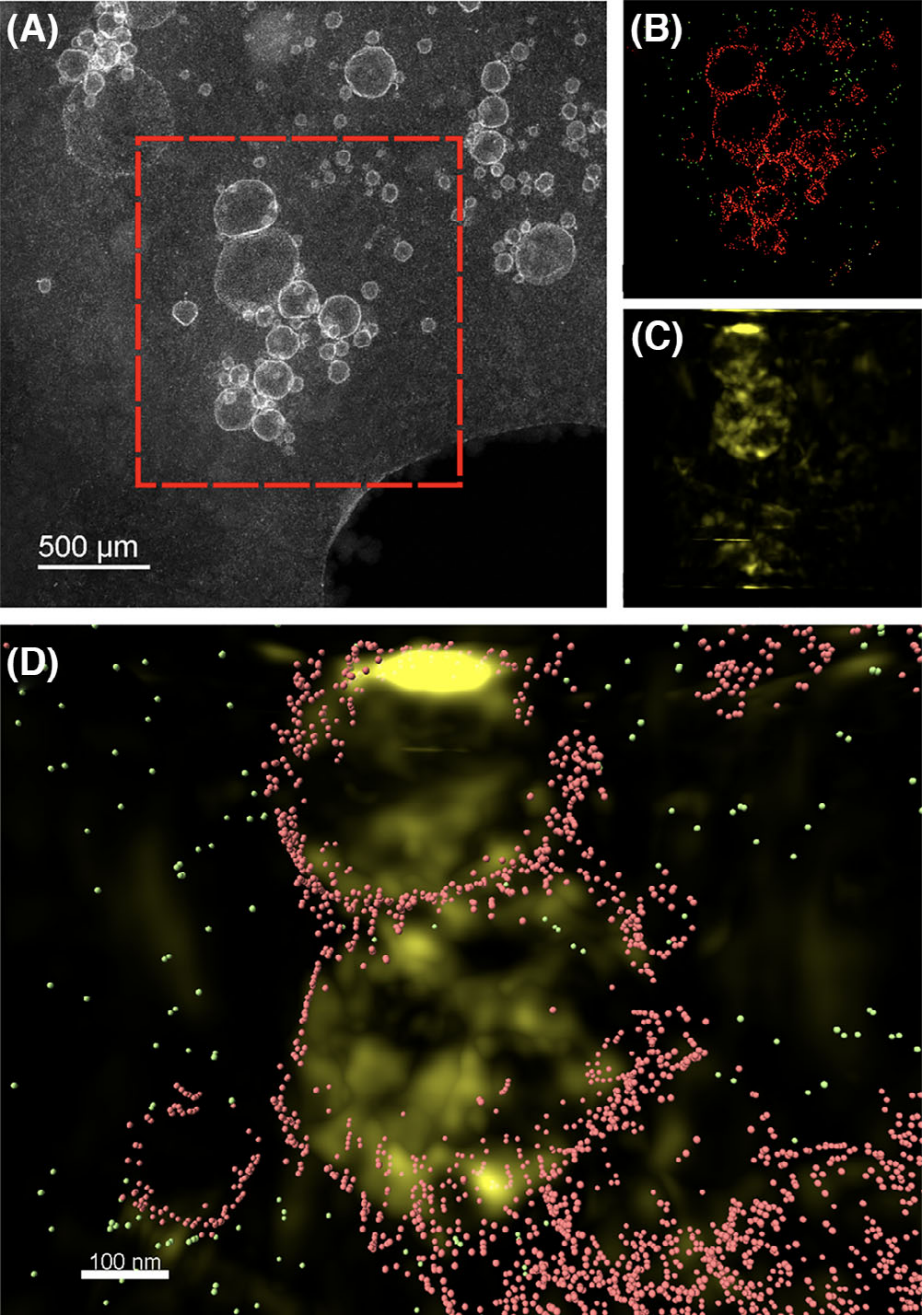
(A) Cryo‐HAADF STEM image of a group of Pickering emulsions and the corresponding tomograms reconstructed from (B) a cryo‐HAADF STEM tilt series and (C) a tilt series of EELS carbon signal (C K edge) maps. (B) The position (in red) of the Pt‐NPs on the oil–water interface and in green Pt‐NPs in the water phase. (D) A magnified superimposition of (B) and (C).

To compensate for the lack of information on the oil phase obtained in the HAADF STEM tomograms, EELS was used as a complementary elemental analysis technique. A total of 9 EELS elemental maps were acquired at intervals alongside the HAADF STEM tilt series (see Videos S2 and S3), making use of the tilt tracking in the Tomography STEM software. After generating elemental maps using the carbon K edge signal in Hyperspy, the EELS tilt series was aligned and reconstructed into a tomogram using the same process outlined in the electron tomography section. The tomograms obtained from the HAADF STEM and EELS signals were then superimposed in Imaris using the HAADF STEM and ADF (collected simultaneously with EELS) supporting images as references (Figure 7). As shown in Figure 7D, the tomogram generated from the carbon K edge map aligns with the approximated position of the oil droplets. The requirement to collect multiple EELS elemental maps drastically increases the total electron fluence needed (by at least a factor of 10), and therefore pixel sizes below 12 nm cannot be used for EELS elemental maps without damage occurring during the tilt series. This limits the use of this technique to larger droplets (>300 nm) and can only give qualitative information on the position of the stabilising nanoparticles. This size limit creates a narrow size range for suitable droplets, with the lower limit being given by the pixel sized needed for EELS and an upper limit given by the sample thickness. It is therefore possible to determine the general position of the particles and whether they are embedded within the oil–water interface, but it is not possible to determine their exact position at the interface (e.g. determine the contact angle for the particles, which is a difficult task for systems where the particles stabilising the interface are <50 nm).

Collecting a single EELS (or EDX) elemental map will cause less electron beam induced damage and be an alternative to provide elemental information in conjunction with a tilt series. In cases where the structures of interest provide clear contrast within the tilt series, it is possible to use EELS or EDX results to aid in segmentation. In the sample presented in Figure 7A the oil phase in the droplet cannot be distinguished from the surrounding ice using cryo‐HAADF STEM imaging alone, and this technique would therefore be unable to provide any 3D information on the oil phase.

### 3.6 Limits to sample analysis and future directions for cryo‐imaging and analysis of Pickering emulsions

Using the current electron microscope setup (detailed in the Materials and Methods), limits in the effective imaging resolution were encountered when attempting to analyse the Pt‐NPs due to the low electron fluence needed to prevent sample damage, with the images collected having low signal‐to‐noise ratios at high magnifications. The solution shown in this study was to simply work at lower magnifications (×20k to ×80k) whenever high total electron fluence was required, as was the case with electron tomography. When using electron tomography with EELS, this approach results in the inability to resolve individual Pt‐NPs on the droplet surface, therefore being unable to analyse their contact angle, which is a characteristic of interest for Pickering emulsions. For the two Pickering emulsions in this study, each with the same Pt‐NPs stabilising a different oil (hexadecane and sunflower oil), it would be interesting to examine the precise location of the stabilising NPs with respect to the oil–water interface, as this effective contact angle measurement is a useful indicator of stability.^31^ An automated cryo‐EM tomography procedure to limit exposure but push resolution to as low as possible could achieve this and such systems are currently being used and developed within other scientific fields. Moving forward applying these techniques to the system analysed here would be the logical next step.^55^, ^64^

Furthermore the use of improved equipment such as a direct electron detection system rather than a CCD camera could help resolve such small NPs during low dose imaging.^55^, ^65^, ^66^ In addition, characterisation of the sample through cryo‐scanning electron microscopy using techniques such as focus ion beam milling would offer a future opportunity to image a larger sample volume and therefore image larger droplet clusters or systems with larger droplet sizes or to select a site of interest to prepare a lamella to cryo‐transfer to the cryo‐(S)TEM.^63^, ^67^, ^68^

## 4 CONCLUSION

Plunge‐freezing was found to be suitable as a preparation technique for Pickering emulsion samples with average droplet sizes below 600 nm for cryo‐(S)TEM imaging while retaining the native structure. At low electron fluences, cryo‐STEM allows for both high‐resolution imaging and elemental analysis using EDX and EELS with minimal beam‐induced damage to the sample. Cryo‐HAADF STEM allowed for easy identification of the Pt‐NPs due to their high atomic number while permitting for a higher total electron fluence before the onset of beam‐induced damage to the sample, thus allowing for imaging at higher resolutions. Although the use of EDX and EELS is limited by the damage caused at high total electron fluences, both techniques remain useful for elemental analysis. EELS can be used to confirm the presence of elements with edges at relatively low electron energy loss (e.g. oxygen, carbon, nitrogen), while EDX permits for the identification of a wider range of elements (including the platinum in the stabilising NPs).

Overall, cryo‐preservation of the Pickering emulsion samples allows for not only cryo‐(S)TEM imaging, but also 3D analysis through cryo‐ET. Tilt series obtained in cryo‐HAADF STEM mode were successful for the creation of 3D tomograms with qualitative information on droplet shape and nanoparticle distribution. Moreover, electron tomography can be combined with EELS to provide 3D information of the oil phase which would otherwise be unattainable in cryo‐HAADF STEM. However, further improvements on the efficiency of the tilt series collection are needed to decrease the total electron fluence, therefore allowing for a potential increase in image resolution to obtain more quantitative data from the 3D tomograms.

## Supporting information



Supporting information

Supporting information

Supporting information

Supporting information
