# Supplementary material for: Native state structural and chemical characterisation of Pickering emulsions: A cryo‐electron microscopy study
Source: J Microsc. 2025 Jan 31;298(1):92–105. doi: 10.1111/jmi.13391 (PMC11891983; doi:10.1111/jmi.13391)
Supplement: Supplementary file 1 — Supporting information [file JMI-298-92-s002.docx]

**Supplementary Information**

**Native state structural and chemical characterisation of Pickering emulsions: A cryo-electron microscopy study**

Dario Luis Fernandez Ainaga, Teresa Roncal-Herrero, Martha Ilett, Zabeada Aslam, Cheng Cheng, James P. Hitchcock, Olivier J. Cayre, Nicole Hondow

School of Chemical and Process Engineering, University of Leeds, Leeds LS2 9JT, United Kingdom

**Materials and Methods**

*Dynamic Light Scattering (DLS)*

A NanoSeries Zetasiser (Malvern Nano-ZS) (DLS) fitted with a He-Ne laser source (633nm wavelength, 4mW power) was used to measure emulsion droplets in suspension.

*Considerations of EELS-ET*

Images for the HAADF STEM tilt series ware collected every 3° in a ±60° range; EELS maps were collected together with the HAADF STEM tilt series at 0°, ±18°, ±27°, ±39°, ±51° angles. A limited number of maps had to be collected to decrease sample damage.

As the tilt angle increases, so does the effective sample thickness the electron beam has to pass through. This has direct effects on the collection of EEL spectra, including an increase in the effects of plural scattering and a lower signal-to-noise ratio. An EELS map was not collected at 60° due to the impracticality of increasing the exposure time or electron dose to increase the signal obtained.

To mitigate these issues, the EELS map at 0° was collected first and used to quickly estimate the sample thickness in the area of interest and detect the presence of carbon film near to or obstructing the sample.

**Figures and Videos**


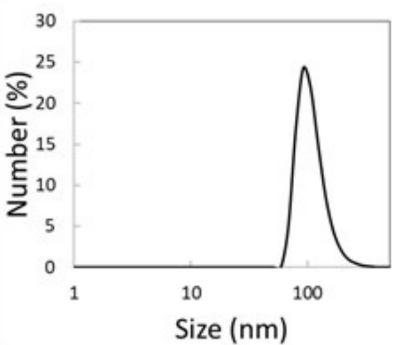


Figure S1. DLS measurements of the sunflower oil Pickering emulsion, with an average diameter of ~100 nm.


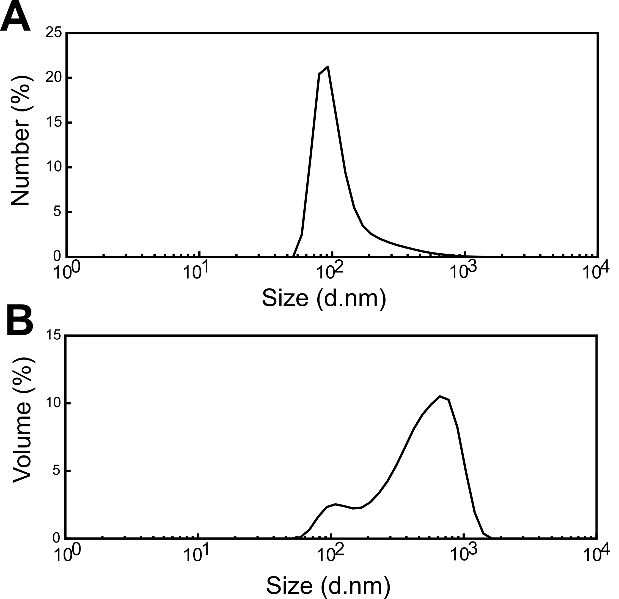


Figure S2. DLS measurements of the hexadecane Pickering emulsion, showing A) the number distribution and B) the volume distribution. The graph in A) shows that similarly to the sunflower oil sample in Figure S1, the hexadecane sample has an average diameter of ~100 nm. The volume distribution graph in B) instead shows two peaks, with a main peak around ~600 nm and a second peak at around ~100 nm.


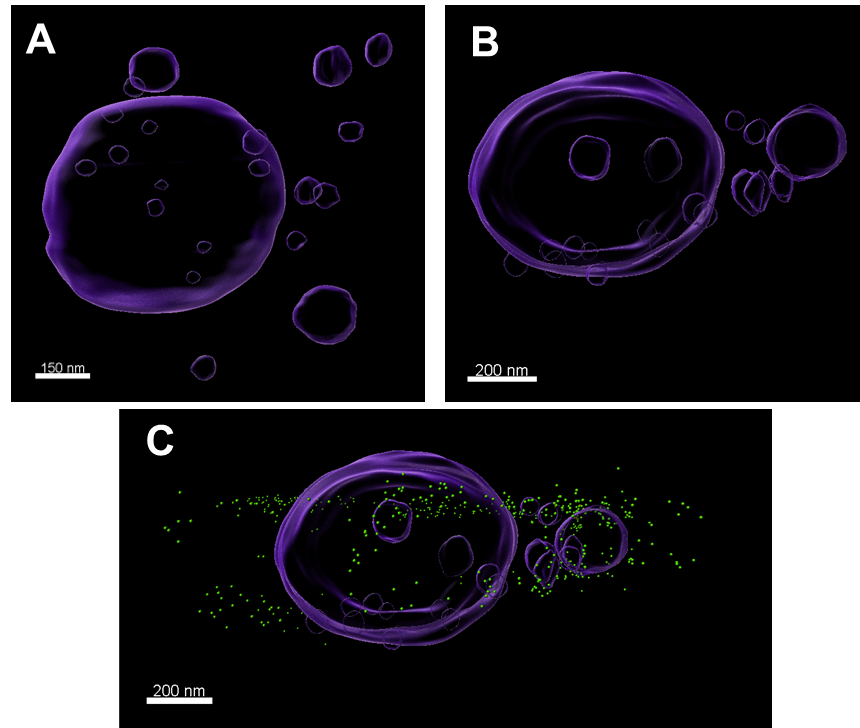


Figure S3. a) Still from the electron tomogram in Figure 4 showing only the approximate shape of the oil droplets and b) 90° tilt view. c) Tomogram with only droplets and Pt-NPs in the water phase. The Pt-NPs in the water phase tend to be found in the water-air interface and seem to suggest that the ice thickness increases near larger oil droplets.


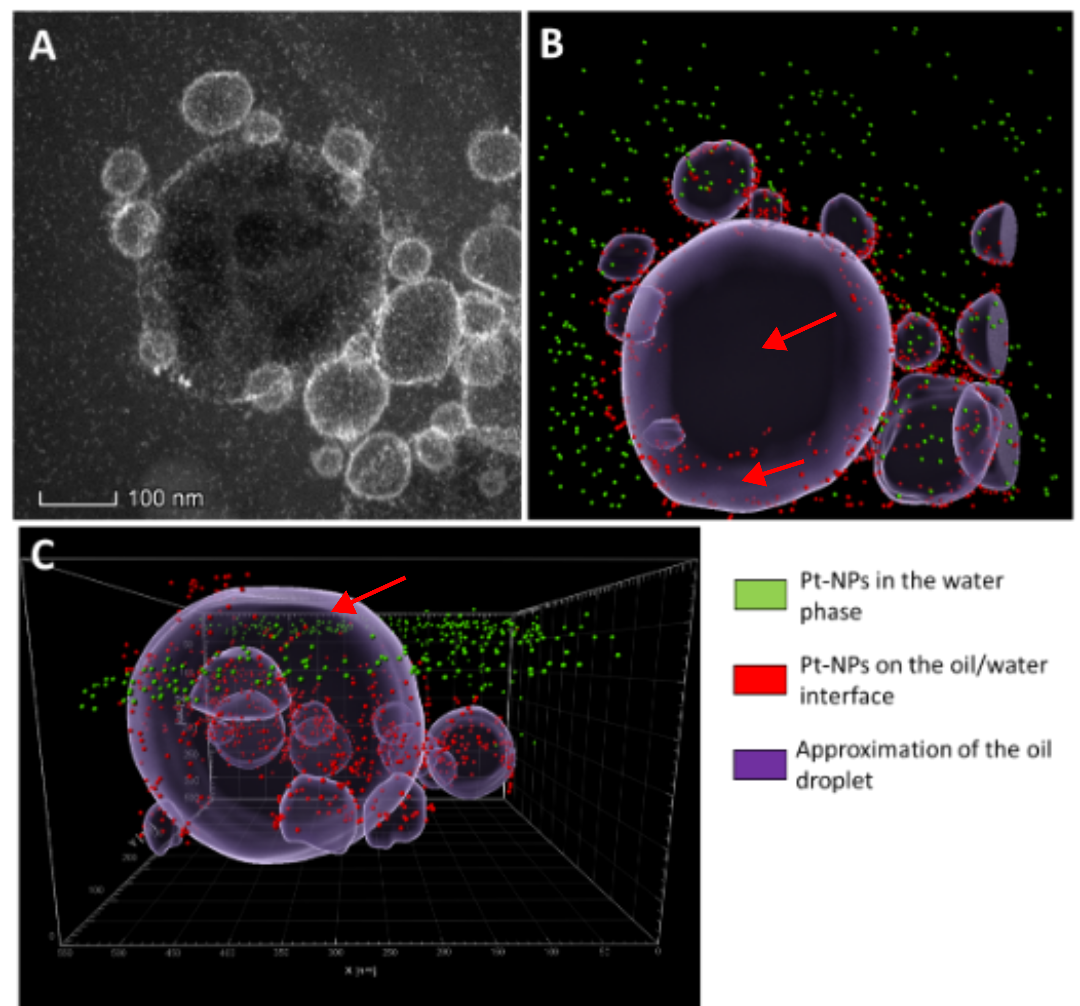


Figure S4. a) Cryo-HAADF STEM image of a droplet cluster and its corresponding tomogram (b-c). The tilt series was taken in a ±60° tilt range, with 5° tilt steps and pixel resolution of 0.63 nm. Despite this attempt to limit damage, signs of ice sublimation are present in the 3D reconstruction, with the droplet shape and position of Pt-NPs being different from that seen in the image taken before the tilt-series (a), especially near the ice surface (indicated by the red arrows).


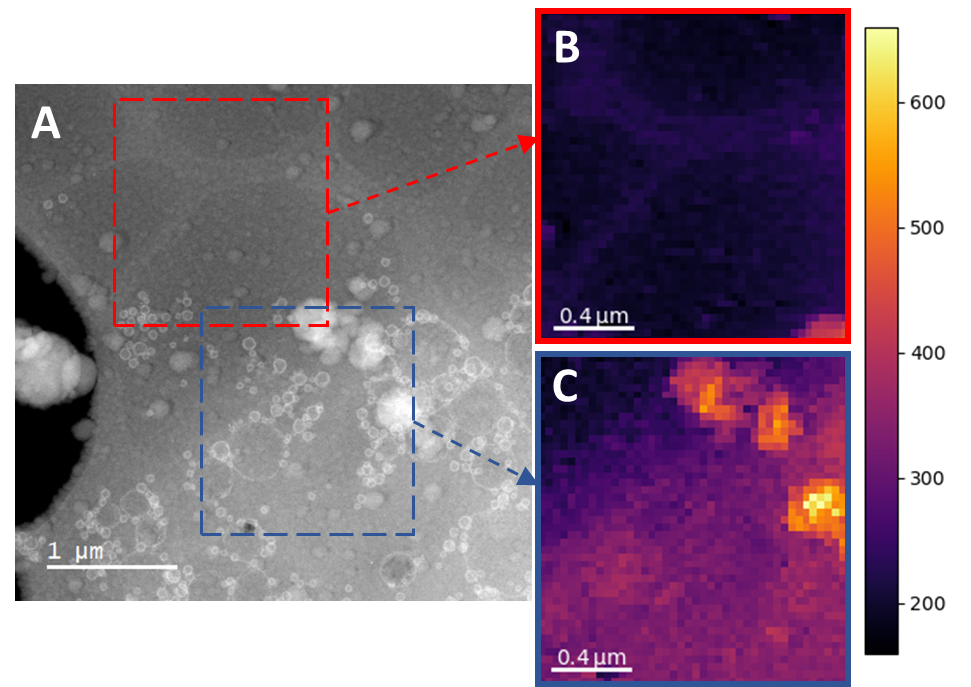


Figure S5. Maps of the estimated absolute ice thickness (in nm) from EEL low-loss spectra taken from two regions of the STEM image (A). The estimated thickness maps for (B) an area without sample (3.97x10^-3^ e^-^/Å^2^) and (C) an area with Pickering emulsion droplets shows that the ice thickness can vary depending on the presence of the sample (0.29 e^-^/Å^2^). Areas containing droplet clusters or larger droplets (>300 nm diameter) will feature thicker ice than what is observed in droplet-free areas.

**SUPPLEMENTARY VIDEO FILES**

| ***File*** | ***Description*** |
| --- | --- |
| Video_SI 1.tif | Aligned and cropped tilt series collected in HAADF STEM mode, corresponding to Figure 4. |
| Video_SI 2.tif | Aligned and cropped tilt series collected in HAADF STEM mode, corresponding to Figure 7. Note the apparent expansion of the droplets as a result of beam-induced damage during the collection of the tilt series and EEL spectra. Especially noticeable movement of the Pt-NPs coincides with the EELS collection steps. |
| Video_SI 3.tif | Series of carbon K edge signal maps used to reconstruct the tomogram in Figure 7. The presence of carbon film in the lower and upper part of the images limited the area that could be successfully reconstructed. |
